# Hemophagocytic Lymphohistiocytosis Associated With T-cell Lymphoma in Pregnancy: Diagnostic Conundrum Unveiled

**DOI:** 10.7759/cureus.66170

**Published:** 2024-08-05

**Authors:** Nor Azlina Siddik, Siti Nur Hidayah Abd Rahim, Jazlan Jamaluddin, Muhamad Yazli Yuhana

**Affiliations:** 1 Family Medicine, Klinik Kesihatan Jinjang, Kuala Lumpur, MYS; 2 Family Medicine, Klinik Kesihatan Taman Ehsan, Kuala Lumpur, MYS; 3 Family Medicine, Klinik Kesihatan Selayang Baru, Selayang, MYS; 4 Infectious Disease, Kumpulan Perubatan Johor (KPJ) Healthcare University, Nilai, MYS; 5 Infectious Disease, Kumpulan Perubatan Johor (KPJ) Ampang Puteri Specialist Hospital, Ampang, MYS

**Keywords:** pyrexia of unknown origin (puo), t-cell lymphoma, hemophagocytic lymphohistiocytosis (hlh), medical disorders in pregnancy, syndrome of fever of unknown origin

## Abstract

Hemophagocytic lymphohistiocytosis (HLH) during pregnancy is a rare and often misdiagnosed disease. The clinical manifestations are non-specific, contributing to a high maternal mortality rate. This case report details the presentation of a 31-year-old pregnant woman with high-grade fever initially treated as an infection-related condition. The diagnostic challenge arose from the rarity of HLH, its variable clinical presentation, and the lack of specificity in clinical and laboratory findings. Despite numerous tests and escalation of therapies, the patient, unfortunately, succumbed to HLH associated with T-cell lymphoma. This case report aims to raise awareness of HLH, emphasizing its challenging definition. Malignancy-associated HLH is not uncommon, and early identification and treatment are paramount to prevent progressive tissue damage, organ failure, and mortality. The atypical presentation of HLH as a clinical manifestation of T-cell lymphoma underscores the need for vigilance in diagnosing this potentially fatal syndrome.

## Introduction

Hemophagocytic lymphohistiocytosis (HLH), also known as hemophagocytic syndrome, is an aggressive and potentially fatal syndrome characterized by over-activation of histiocytes and lymphocytes [1]. While typically seen in infancy, secondary HLH can occur in adults, triggered by various factors disrupting immune homeostasis, with infections being a common instigator [2]. Malignancies, autoimmune diseases, metabolic disorders, and acquired immune deficiencies also contribute to secondary HLH [3].

Malignancy-associated HLH (M-HLH), particularly hematologic neoplasms, has gained attention, with an increased incidence of B- and T-cell lymphomas. HLH affects 1% of adults with hematologic tumors, although in certain patients with B- and T-cell lymphomas, the incidence has risen to 20% [4]. A large series of studies from 2,197 adult HLH patients have demonstrated that M-HLH accounts for almost 50% of adult HLH. The most common tumor types causing HLH are hematological neoplasms (93.7%), specifically T- or natural killer (NK) cell lymphoma or leukemia (35.2%), followed by B-cell lymphoma (31.8%), other unspecified hematologic neoplasms (14.4%), leukemia (6.4%), and Hodgkin lymphoma (5.8%) [5]. Solid tumors and non-specified neoplasms account for 3.1% and 3.2% of all neoplasms, respectively. M-HLH typically presents with symptoms that overlap with other types of HLH, leading to a higher incidence of misdiagnosis and mortality. Impaired function of cytotoxic T lymphocytes and NK cells, as well as macrophages, has resulted in HLH clinical features such as prolonged fever, hepatosplenomegaly, cytopenia, hypertriglyceridemia, hyperferritinemia, and hemophagocytosis in bone marrow, liver, spleen, or lymph nodes [6].

While malignancy is rare in pregnant women due to their overall age group, HLH can serve as an initial symptom of hematologic tumors. Lymphomas, the most prevalent blood tumors during pregnancy, have an incidence rate of 1:6000. Therefore, when an expectant mother receives an HLH diagnosis, prompt exclusion of lymphoma and other tumors is vital due to their poor prognosis, necessitating early treatment [7]. HLH can arise at any stage of pregnancy, with a mortality rate exceeding 80%. A study has shown that approximately half of the cases occur within three days, and three-quarters occur within 10 days after delivery, potentially related to postpartum physiological fluctuations and infection [7]. About 3.7% of patients with malignancy present with HLH, suggesting that it may act as the first symptom of hematologic tumors during pregnancy. The median survival time in M-HLH is two months. The lack of awareness contributes to delayed recognition and treatment, emphasizing the need for increased vigilance among healthcare professionals [8].

## Case presentation

A 31-year-old Chinese lady, primigravida, was receiving routine antenatal care at a health clinic with uncomplicated antenatal follow-ups. This pregnancy was planned after being married for two years. She received two doses of the CoronaVac COVID-19 vaccine at three weeks and 18 weeks of pregnancy with a booster dose of BNT162b2 at 32 weeks of gestation. However, at 34 weeks gestation, she presented with increasing lethargy, headache, and high-grade fever for one week. On review of systems, there was no cough, hemoptysis, diarrhea, or traveling history. Her temperature was 39^o^C with blood pressure of 112/61 mmHg and heart rate of 113 beats per minute. Her respiratory rate was 20 breaths per min with an oxygen saturation of 98% under room air. The systemic examination was unremarkable. Her abdominal examination revealed normal pregnancy-related signs with no palpable spleen or liver. There were no cervical or inguinal lymph nodes palpable. Initial investigations showed leukocytosis of 19.3 x10^9^/L with significant anemia from baseline hemoglobin of 11.5 g/dL (Table 1). A chest X-ray showed pneumonia in bilateral lower zones with right pleural effusion. No further imaging was done. She was admitted to the hospital with the preliminary diagnosis of pneumonia and started on oral amoxicillin/clavulanic acid. As her fever persisted, she was switched to intravenous (IV) cephalexin. Initial urine and blood culture showed no growth. Further tests for dengue, influenza, COVID-19, Mycoplasma pneumonia, leptospirosis, typhoid, malaria, and anti-nuclear antibody were negative.

**Table 1:** Summary of the patient’s investigations during hospital admission.

Investigations/Admission Day	1	4	7	10	11	12	13	14	15	16	17
Hemoglobin (g/dL)	9.7	9.1	10.8	10.0	8.0	10.0	7.7	9.8	10.2	9.0	8.0
White-cell count (x10^9^/L)	19.3	13.6	19.6	29.1	59.6	41.6	47.4	46.0	53.2	73.4	86.4
Neutrophils (%)	89.3	91.4	85.5								
Lymphocytes (%)	4.5	4.0	9.6								
Platelet count (10^3^/μL)	197	174	174	74	128	54	52	45	42	47	130
Urea (mmol/L)	2.5		2.6								
Sodium (mmol/L)	129		129								
Potassium (mmol/L)	3.7		2.9								
Chloride (mmol/L)	100		99								
Creatinine (μmol/L)	51	-	49	54	82	70	144	189	139	133	70
Alanine transaminase (g/dL)	72	-	52	51	85	28	26	29	36	40	28
Aspartate transaminase (g/dL)	88	-	58	44	31	106	119	145	225	270	106
Ferritin (*μ*g/L)	-	-	503	-	-	-	4310	6057	10440		37451
C-reactive protein	<6.0	82	107.1	-	-	-	-	-	-	-	-
Lactate dehydrogenase (U/L)	-	-	341	-	-	-	550	-	-	-	-
Procalcitonin (ng/mL)	-	-	5	18	-	25	25	50	22	17	-
Fibrinogen (mg/dL)	-	-	-	-	2.3	1.9	1.0	1.3	-	-	-

After a week, her condition showed no improvement despite being on antibiotics, leading to a referral to an infectious disease physician for pyrexia of unknown origin. At the time, she appeared lethargic but alert, and attentive. Physical examination revealed reduced breath sounds in both lungs and increased vocal resonance in the lower right zone, but no cervical lymph nodes were palpable. She was treated for nosocomial pneumonia with sepsis and received IV cefepime after one week of IV cephalexin.

Within 48 hours, the antibiotic was changed to IV meropenem and cefazolin since there was no improvement. Further investigations showed an increasing white cell count from 21 up to a peak of 29.1x10^9^/L, along with moderate anemia and worsening thrombocytopenia. Procalcitonin and ferritin were increased, but fibrinogen levels were normal. The elevated procalcitonin prompted further investigations and treatment for infection, but no microorganisms were detected in the tests. Computed tomography (CT) of the chest showed multiple enlarged mediastinal, para-aortic, and pelvic lymph nodes with bilateral pleural effusion and collapse consolidation at the right lower lobe. CT abdomen revealed the presence of multiple abdominal lymph nodes, hepatosplenomegaly, and an enlarged thymus. The patient was empirically treated for extrapulmonary tuberculosis while lymphoma was ruled out. At 35 weeks gestation on day 10 of admission, the patient had a spontaneous vaginal delivery of a healthy baby boy weighing 2.5 kg.

**Figure 1:**
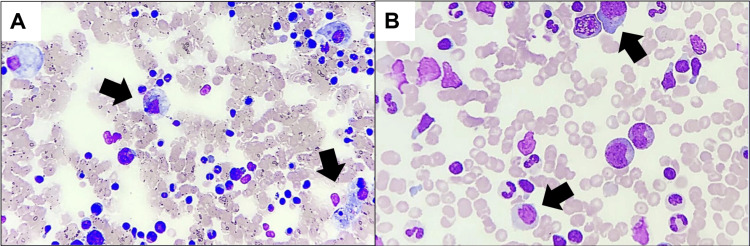
Bone marrow aspirate slides showing an increase in lymphocytosis and hemophagocytic activity (A) with lymphoid with blebs and (B) May-Grünwald–Giemsa stained, 20x magnification.

**Figure 2:**
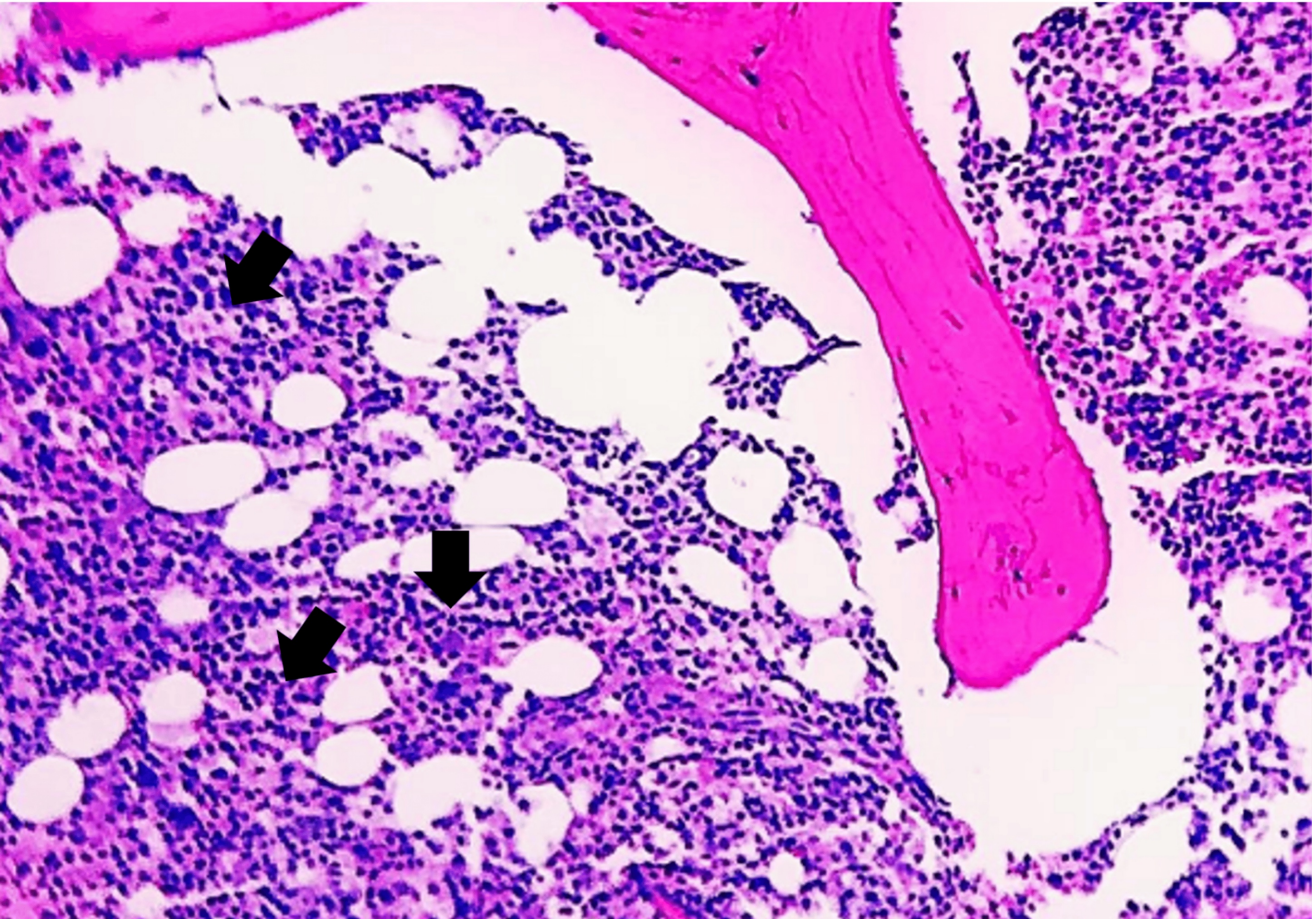
Trephine biopsy with hypercellular bone marrow with diffuse infiltration of abnormal lymphoid cells (Hematoxylin and eosin stained, 20x magnification).

Further tests for tuberculosis, including pleural tapping, came back negative. Bone marrow and trephine (BMAT) biopsy were also performed (Figure 1). However, the patient's condition deteriorated on the second day after delivery, leading to multiple organ failure. Blood tests showed metabolic acidosis and high anion gap acidosis. HLH was suspected after consultation with a hematologist, and treatment with IV dexamethasone and etoposide was initiated. She continued to deteriorate needing intubation for airway protection. Antimicrobials were also escalated with the addition of IV ciprofloxacin and anidulafungin. Continuous renal replacement therapy was also started due to persistent metabolic acidosis and high anion gap acidosis. Unfortunately, the patient did not respond to the treatment and passed away four days later. The final results of the BMAT biopsy reported lymphomatous infiltration and an increase in hemophagocytic activity likely due to an underlying lymphoid malignancy (Figure 2). A posthumous diagnosis of T-cell lymphoma in the leukemic phase with HLH and multiorgan failure was established.

## Discussion

HLH during pregnancy poses a complex diagnostic challenge, primarily due to its rarity, the myriad of clinical presentations, and the lack of specificity in clinical and laboratory findings. To the best of our knowledge, only a small number of HLH and lymphoma cases have been documented, due to the low incidence of HLH and lack of knowledge [7]. In this case, the initial presentation of high-grade fever, lethargy, and headache led to an empirical diagnosis of pneumonia, supported by chest x-ray findings. However, the lack of clinical improvement prompted a shift in diagnostic focus towards HLH. A pivotal aspect in the diagnostic odyssey was the elevated procalcitonin levels, a biomarker recognized for its association with malignancy and infection. While procalcitonin played a crucial role in prompting further investigations and ruling out infectious causes, its limited accessibility in all tertiary centers raises concerns [9]. Wider availability of such biomarkers would likely enhance the early identification of HLH, especially in cases where the clinical presentation overlaps with infectious etiologies. Early imaging, particularly CT scans, could have been instrumental in this case [10]. The detection of lymphadenopathy and hepatosplenomegaly through CT earlier in the diagnostic process could have provided critical breakthrough findings. The potential role of magnetic resonance imaging (MRI) should also be considered in persistent culture-negative sepsis-like features, as it may offer additional diagnostic clarity.

The subsequent empirical treatment for extrapulmonary tuberculosis further exemplifies the diagnostic dilemma. The overlap of symptoms between HLH and other infectious or inflammatory conditions, compounded by the lack of specific diagnostic tests, contributes to delays in implementing targeted therapies [1]. This diagnostic uncertainty is particularly pronounced in smaller healthcare institutions, where fulfilling the HLH diagnostic criteria, requiring the presence of five out of eight specific parameters, poses a substantial challenge. In response to these challenges, Tamamyan et al. have proposed a broader diagnostic category to heighten suspicion of HLH [8]. This includes parameters such as hepatic or renal failure of unknown etiology, sudden onset multi-organ failure, culture-negative sepsis, and encephalopathy of unknown origin. In our case, the patient's symptoms aligned with these broader criteria, suggesting that early application of this approach could have facilitated an earlier diagnosis and timely intervention. Incorporating these expanded diagnostic criteria in clinical practice could help reduce the threshold for considering HLH as a potential diagnosis, especially in resource-limited settings.

Despite the initiation of dexamethasone and etoposide, the patient's prognosis remained poor, consistent with the aggressive clinical course frequently observed in M-HLH [4,11]. The poor prognosis in malignancy-associated HLH is compounded by the advanced disease stage where initial effective therapy is not effective and immunocompromised status whereby cytotoxic therapy is not an ideal option for the patient [8]. The delayed diagnosis and treatment initiation, inherent to the challenges in recognizing HLH, significantly impact patient outcomes. Additionally, it is noteworthy that the patient received a COVID-19 booster two weeks before the onset of symptoms. While the relationship between COVID-19 mRNA vaccines and HLH is not well-established, there have been reports suggesting a potential link [12,13]. Most of them are due to secondary causes well known to cause HLH, such as EBV infection and malignancy. Hyperinflammatory syndrome, possibly triggered by recent vaccinations such as T-cell activation, has been implicated in the development of HLH [14]. There is a need for further investigation into this association. Therefore, it is important to consider recent vaccination history in the diagnostic process.

This case also underscores the necessity of timely intervention with more advanced investigations, such as CT scan or MRI, when fever does not respond to traditional antibiotics and cultures remain negative. The frequent switching of antibiotics without substantial reasoning should be highlighted as a point of caution. This case exemplifies the importance of multidisciplinary management in such complicated cases during pregnancy, which is key to comprehensive care. This approach is emphasized in multiple confidential inquiry reports on maternal death, highlighting the critical need for coordinated care involving various specialties to improve patient outcomes. This case also highlights the urgent need for education and awareness campaigns targeting healthcare practitioners to enhance clinical suspicion and promote timely intervention. Additionally, research into expanding the availability of biomarkers like procalcitonin, coupled with the incorporation of broader diagnostic parameters, may offer a more nuanced approach to identifying and treating HLH in diverse clinical settings. Through these efforts, hopefully, the diagnostic challenges posed by HLH during pregnancy can be mitigated and, ultimately, improve patient outcomes.

## Conclusions

This case report highlights the critical need for heightened awareness, timely diagnosis, and early intervention of HLH during pregnancy, especially in the context of malignancy. Early and precise identification is paramount to preventing progressive tissue damage, organ failure, and mortality. Our case demonstrates the importance of considering advanced imaging techniques, such as MRI, when fever does not respond to traditional antibiotics and cultures remain negative. The frequent switching of antibiotics without substantial justification emphasizes the necessity for a more structured diagnostic approach.

The atypical presentation of HLH as a clinical manifestation of T-cell lymphoma underscores the need for vigilance in diagnosing this potentially fatal syndrome. Multidisciplinary management of such complicated cases during pregnancy is essential, as highlighted in multiple confidential inquiry reports on maternal death. Coordination among various specialties is key to comprehensive care and improved patient outcomes. By addressing these points, healthcare professionals can better navigate the diagnostic challenges posed by HLH, ultimately enhancing patient care and prognosis.
